# Cefepime and Amoxicillin Increase Metabolism and Enhance Caspofungin Tolerance of *Candida albicans* Biofilms

**DOI:** 10.3389/fmicb.2019.01337

**Published:** 2019-06-28

**Authors:** Rossana de Aguiar Cordeiro, Antonio Jose de Jesus Evangelista, Rosana Serpa, Ana Raquel Colares de Andrade, Patrícia Bruna Leite Mendes, Jonathas Sales de Oliveira, Lucas Pereira de Alencar, Vandbergue Santos Pereira, Reginaldo Gonçalves Lima-Neto, Raimunda Nogueira Brilhante, José Júlio Costa Sidrim, Débora Castelo Brancode Souza Collares Maia, Marcos Fábio Gadelha Rocha

**Affiliations:** ^1^Medical Mycology Specialized Center, Federal University of Ceará, Fortaleza, Brazil; ^2^Department of Tropical Medicine, Federal University of Pernambuco, Recife, Brazil; ^3^Post Graduate Program in Veterinary Sciences, College of Veterinary Medicine, State University of Ceará, Fortaleza, Brazil

**Keywords:** β-lactam antibiotics, cefepime, amoxicillin, *Candida albicans*, biofilm

## Abstract

It is well known that prolonged antibiotic therapy alters the mucosal microbiota composition, increasing the risk of invasive fungal infection (IFI) in immunocompromised patients. The present study investigated the direct effect of β-lactam antibiotics cefepime (CEF) and amoxicillin (AMOX) on biofilm production by *Candida albicans* ATCC 10231. Antibacterials at the peak plasmatic concentration of each drug were tested against biofilms grown on polystyrene surfaces. Biofilms were evaluated for biomass production, metabolic activity, carbohydrate and protein contents, proteolytic activity, ultrastructure, and tolerance to antifungals. CEF and AMOX enhanced biofilm production by *C. albicans* ATCC 10231, stimulating biomass production, metabolic activity, viable cell counts, and proteolytic activity, as well as increased biovolume and thickness of these structures. Nevertheless, AMOX induced more significant changes in *C. albicans* biofilms than CEF. In addition, it was shown that AMOX increased the amount of chitin in these biofilms, making them more tolerant to caspofungin. Finally, it was seen that, in response to AMOX, *C. albicans* biofilms produce Hsp70 – a protein with chaperone function related to stressful conditions. These results may have a direct impact on the pathophysiology of opportunistic IFIs in patients at risk.

## Introduction

Invasive fungal infections (IFIs) are a serious public health problem worldwide, as they result in approximately 90% mortality in immunocompromised patients, such as severe neutropenic transplant patients using high doses of corticoids and HIV-positive individuals (Hahn-Ast et al., 2010; Mariette et al., 2017). Such infections are usually aggravated when biofilm-forming pathogens are involved (Vogel et al., 2013; Johnson et al., 2016). Prolonged antibiotic therapy, parenteral nutrition, chemotherapy, and breakdown of cutaneous–mucosal barriers are important determinants for the development of such biofilms (Ramage et al., 2009; Spitzer et al., 2017).

It is known that broad-spectrum antibacterials alter mucosal microbiota composition, eliminating many bacterial cells, hence allowing the colonization of these sites by *Candida* spp. (Samonis et al., 2013). Under such circumstances, *Candida albicans* may be privileged: lipopolysaccharide molecules, which are important immunomodulators present in bacterial cell wall, can directly react with the fungal cells, increasing its virulence (Rogers et al., 2013). A previous study has shown that bacterial peptidoglycan subunits stimulate the yeast-to-hyphae transition in *C. albicans*, helping the pathogen to escape from the immune system (Xu et al., 2008). Broad-spectrum antibacterials, such as β-lactams, eliminate bacterial microbiota and increase the amount of such peptidoglycan subunits in serum, thus enhancing the risk of opportunistic fungal infections (Wang, 2013). Thus, antibiotic therapy is a determining risk factor for the development of IFI in immunocompromised patients (Bongomin et al., 2017).

Thus, the present study evaluated the direct effect of two broad-spectrum antibiotics, cefepime (CEF) and amoxicillin (AMOX), on *C. albicans* biofilms. We investigated if antibiotics could directly enhance *C. albicans* biofilm production and metabolism and alter biofilm antifungal susceptibility.

## Materials and Methods

### Microorganism and Antibiotics

The research was carried out with *C. albicans* ATCC 10231. We tested two β-lactam antibiotics commonly used for the treatment of bacterial infections in neutropenic patients with hematological malignancies (Gustinetti and Mikulska, 2016): CEF (Novafarma, Anápolis, Brazil) at 126 μg/ml and AMOX (Sigma-Aldrich, MO, United States) at 4 μg/ml. These values correspond to the respective peak plasmatic concentration (PP) of each drug (Brunton et al., 2018). Stock solutions were diluted in sterile distilled water according to the manufacturer’s recommendations.

### Effect of CEF and AMOX on Biomass, Metabolic Activity, Viable Cells, and Quantification of Carbohydrates and Proteins of *C. albicans* Biofilm

The effect of CEF and AMOX on biofilm production by *C. albicans* ATCC 10231 was performed according to Cordeiro et al. (2015). The biofilms of *C. albicans* ATCC 10231 were formed in 96-well flat bottom microtiter plates with an initial inoculum of approximately 3 × 10^6^ cells/ml in RPMI-1640 medium supplemented with CEF or AMOX. The plates were incubated at 37°C and analyzed at 6, 24, and 48 h of incubation for biomass production, metabolic activity (Cordeiro et al., 2015), and viable cells (Cordeiro et al., 2017). Controls were conducted in RPMI medium without antibiotics; assays were performed in triplicate at two independent experiments.

The effect of CEF and AMOX on *C. albicans* ATCC 10231 biofilm composition was evaluated by staining with 1% calcofluor-white (Sigma-Aldrich, MO, United States) (Clark et al., 2018), 0.1% Congo Red (Sigma-Aldrich, MO, United States) (Bazzini et al., 2011), and 0.1% safranin (Sigma-Aldrich, MO, United States) (Anne-Marie et al., 2014) for carbohydrates, and SYPRO^®^Ruby (Thermo Fisher Scientific, NY, United States) (Mohammed et al., 2013) for proteins. Biofilms were formed on microplates as previously described. After 48 h of incubation in RPMI medium supplemented with CEF or AMOX, the supernatant was aspirated. Adhered cells were washed twice with sterile PBS and stained with the dyes cited above. Fluorescence readings at 430 nm/510 nm and 465 nm/630 nm were performed on Cytation 3 equipment (BioTek, VT, United States) for calcofluor-white and SYPRO^®^Ruby staining, respectively. For Congo Red and safranin, readings were performed in a spectrophotometer (Celer Biotecnologia S/A, Minas Gerais, Brazil) at 490 and 630 nm, respectively. Controls were conducted in RPMI medium without antibiotics; experiments were performed in triplicate at two independent experiments.

### Effect of CEF and AMOX on the Proteolytic Activity of *C. albicans* Biofilm

The proteolytic activity was performed according to Cordeiro et al. (2017). Biofilms were assembled as previously described. At 6, 24, and 48 h of incubation, an aliquot of 200 μl of biofilm supernatant was collected and added to 200 μl of 0.3% azoalbumin solution (diluted in 1% sodium bicarbonate solution, pH 8.3) and then incubated in a water bath at 37°C for 3 h. Enzymatic reaction was stopped with 5% trichloroacetic acid, followed by the addition of 0.5 M NaOH. Readings were performed at 440 nm in a spectrophotometer. Controls were performed in medium without fungal cells (blank) and also in RPMI medium with microorganisms and without the drugs. Assays were performed in triplicate at two independent experiments.

### Effect of CEF and AMOX on Morphology and Ultrastructure of Biofilms Produced by *C. albicans*

The effect of the CEF and AMOX on the morphology and ultrastructure of the *C. albicans* ATCC 10231 biofilm was evaluated by scanning electron microscopy (SEM) (Cordeiro et al., 2017) and confocal microscopy (CLSM) (Kagan et al., 2014). For both analyses, biofilms were formed on Thermanox^®^slides (Thermo Fisher Scientific, NY, United States) with an initial inoculum of 3 × 10^6^ cells/ml in RPMI-1640 medium at 6, 24, and 48 h of incubation.

For SEM analysis, after incubation for 48 h at 37°C, the biofilms were fixed with 2.5% glutaraldehyde in 0.15 M sodium cacodylate buffer containing alcian blue (0.1%) and incubated overnight at 4°C. Thereafter, the biofilms were washed twice with 0.15 M cacodylate buffer for 5 min and subjected to increasing alcoholic dehydration in ethanol [50, 70, 80, 95, and 100% (twice), 10 min each]. Subsequently, slides were dried with hexamethyldisilazane (Polysciences Europe, Germany) for 30 min and then incubated overnight in a desiccator. Thermanox^®^slides were coated with 10 nm gold (Emitech Q150T, Lewes, United Kingdom) and observed in a SEM (FEI Inspect S50, OR, United States) in the high vacuum mode at 15 kV. For CLSM, biofilms were stained with the Live/Dead^TM^ (Invitrogen, CA, United States) viability kit and evaluated with a confocal Nikon C2 + microscope (Nikon, NY, United States) at 488 nm for the detection of SYTO 9 and at 561 nm for the detection of propidium iodide. The images were acquired from a series of horizontal (*x*–*y*) optical cuts with a thickness of 2.5 μm with intervals of 0.62 μm, along the biofilm depth. For the analysis of the images, five random points equidistant were selected from the three-dimensional biofilm images for biovolume determination and thickness based on the Z-slice using ImageJ 1.50i software (Collins, 2007; Clark et al., 2018).

### Effect of CEF and AMOX on the Composition of the Biofilm Matrix Produced by *C. albicans*

The effect of CEF and AMOX on the matrix protein composition of *C. albicans* biofilms was determined by matrix-assisted laser desorption/ionization time-of-flight mass spectrometry (MALDI-TOF MS). For this purpose, biofilms were formed as described previously for 48 h. Then, biofilms were scraped with sterile tips and the biomass was sonicated. The material was then collected and centrifuged at 9,167 ×*g* for 10 min; supernatants were filtered in 0.22-μm membranes and lyophilized for further analysis (Costa et al., 2012).

Matrix proteins were dissolved in 0.1% trifluoroacetic acid (v/v) in high performance liquid chromatography (HPLC) water solution. One microliter of matrix protein solution was directly spotted in duplicate on polished steel target plate (Bruker Daltonics, Bremen, Germany) and overlaid with α-cyano-4-hydroxycinnamic acid (CHCA) matrix solution [75 mg ml^-1^ of CHCA matrix in ethanol/deionized water/acetonitrile (1:1:1) with 0.03% trifluoroacetic acid] and dried. For mass spectrum acquisition, a MALDI-TOF MS Auto flex III mass spectrometer (Bruker Daltonics, MA, United States) equipped with a 1064-nm Nd : YAG laser (positive reflector mode and laser frequency of 100 Hz) was used with detection range *m*/*z* 400–5,000. Peptide calibration standard I (Bruker Daltonics, MA, United States) was used for calibration (Cordeiro et al., 2017).

Spectra obtained from different treatments were compared by using local Open Source Mass Spectrometry Toll named mMass software version 5.5.0^1^ (Prague, Czechia). Before, to compare pick list, the data processing was performed by Peak Picking, Baseline Correction and Smoothing, according to Niedermeyer and Strohalm (2012). Afterward, selected parent ions were fragmented using LIFT mode. Data were acquired using the Flex Control software and spectra were processed using Flex Analysis software (both Version 3.3, Bruker Daltonics), according to Biemann (1992).

Spectra were putatively identified by using SearchGUI software (Compomics Inc.) methods of peptide mass fingerprinting for MS data and ion search for MS/MS data, against a *C. albicans* ATCC MYA-2876 protein database obtained from UniProt^2^ (Hinxton, United Kingdom). To visualize and analyze the SearchGUI results, a search engine independent platform of peptide and protein identification named PeptideShaker was used (Vaudel et al., 2011). Putative annotated protein sequences were retrieved in FASTA format from the UniProt access^3^.

### Effect of CEF and AMOX on the Antifungal Tolerance of *C. albicans* Biofilm

The effect of CEF and AMOX on the antifungal tolerance of *C. albicans* ATCC 10231 biofilms was evaluated according to Ferreira et al. (2009), with adaptations. Biofilms were formed in 96-well microplates in RPMI medium supplemented with CEF or AMOX, as previously described. After 48 h of biofilm maturation at 37°C, the supernatant was aspirated and the biofilms were washed twice with sterile PBS. Then, a newly fresh 200-μl aliquot of RPMI medium supplemented with antifungals was added to the wells at the following concentrations: amphotericin B (Sigma-Aldrich, MO, United States) at 0.5 and 5 μg/ml; fluconazole (Pfizer, Sao Paulo, Brazil) and itraconazole (Janssen Pharmaceutical, Beerse, Belgium) at 0.25 and 2.5 μg/ml; voriconazole (Pfizer, Sao Paulo, Brazil) at 0.125 and 1.25 μg/ml; and caspofungin (Sigma-Aldrich, MO, United States) at 0.125 and 1.25 μg/ml. Concentrations correspond to minimum inhibitory concentration (MIC) and 10 × MIC of each drug, respectively, against *C. albicans* ATCC 10231 planktonic cells. After 48 h of incubation at 37°C, biofilms were evaluated for biomass production and metabolic activity (Cordeiro et al., 2017). The tests were performed in triplicate at two independent experiments.

### Statistical Analysis

Results were evaluated by analysis of variance (ANOVA), and the means of the data were compared by the Tukey post-test. For all evaluations, *P* values lower than 0.05 were considered significant. Statistical analyses were performed using GraphPad Prism^®^7.0 software (GraphPad Software, CA, United States).

## Results

**FIGURE 1:**
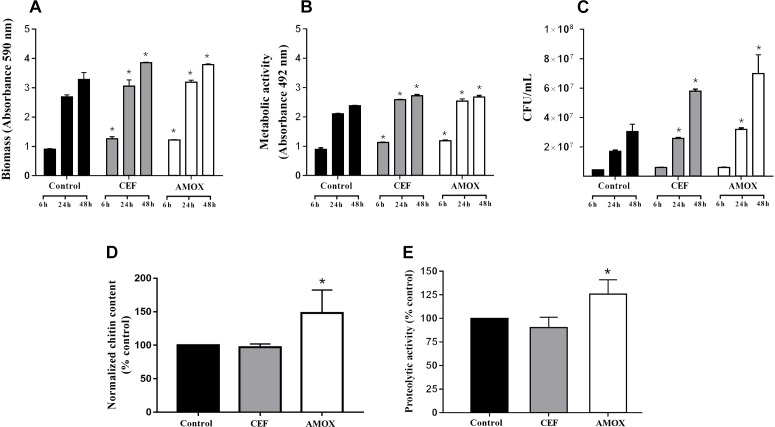
Effect of CEF and AMOX on *C. albicans* ATCC 10231 biofilm regarding to biomass **(A)**, metabolic activity **(B)**, viable cell counts **(C)**, chitin content normalized per biomass **(D)**, and proteolytic activity **(E)**. Black bars: drug-free control; gray bars: treatment with CEF; white bars: treatment with AMOX. Panels **(A–C)** show analyses of 6-, 24-, and 48-h-grown biofilms. Panels **(D,E)** show analyses of 48-h-grown biofilms. Experiments were conducted in triplicate. ^∗^Statistically significant differences when compared to the respective drug-free control (*P* < 0.05), according to ANOVA and Tukey test.

Cefepime and AMOX stimulated biomass production (Figure 1A) and metabolic activity (Figure 1B) of *C. albicans* ATCC 10231 biofilms at 6, 24, and 48 h (*P* < 0.05) of incubation and the number of viable cells (Figure 1C) at 24 and 48 h (*P* < 0.05). In addition, AMOX increased the amount of chitin in *C. albicans* ATCC 10231 biofilms after 48 h of maturation (Figure 1D) (*P* < 0.05). However, antibiotic treatment did not change the amount of proteins in biofilms (data not shown). Treatment with AMOX increased proteolytic activity (Figure 1E) only in 48-h-grown biofilms (*P* < 0.05). There were no differences in proteolytic activity at 6 and 24 h of incubation.

**FIGURE 2:**
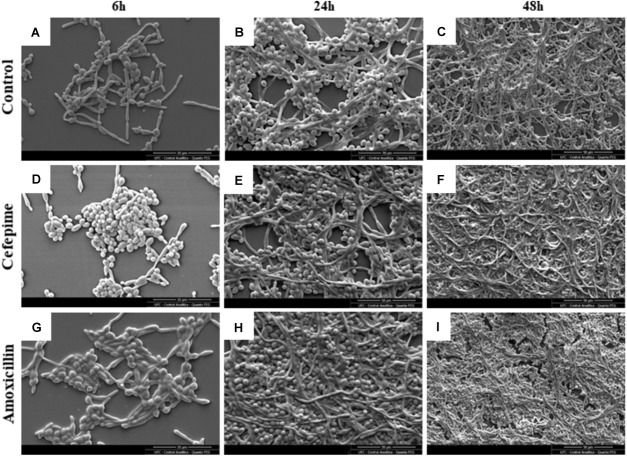
Scanning electron microscopy images of *C. albicans* ATCC 10231 biofilms formed in RPMI medium without antibiotics (drug-free control) **(A,D,G)** or supplemented with CEF **(B,E,H)** or AMOX **(C,F,I)**. Magnification: 2,000×.

Scanning electron microscopy did not allow the detection of substantial differences between drug-free and antibiotic-treated biofilms, except for higher density in CEF- and AMOX-treated biofilms. Analyses revealed microcolonies formed by blastoconidia and pseudohyphae adhered to the substrate after 6 h of incubation (Figure 2A,D,G). Twenty-four-grown biofilms showed an increase in the amount of blastoconidia and adhered filaments, forming dense and organized structures arranged in cellular multilayers, surrounded by an incipient extracellular material (Figure 2B,E,H). At 48 h, biofilms showed a higher cell density, mainly composed of blastoconidia, pseudohyphae, and true elongated hyphae, enclosed by a dense extracellular material, and irregularly interrupted by many channels (Figure 2C,F,I). Mature biofilms formed in RPMI supplemented with AMOX showed patches of dense material under fragmentation.

**FIGURE 3:**
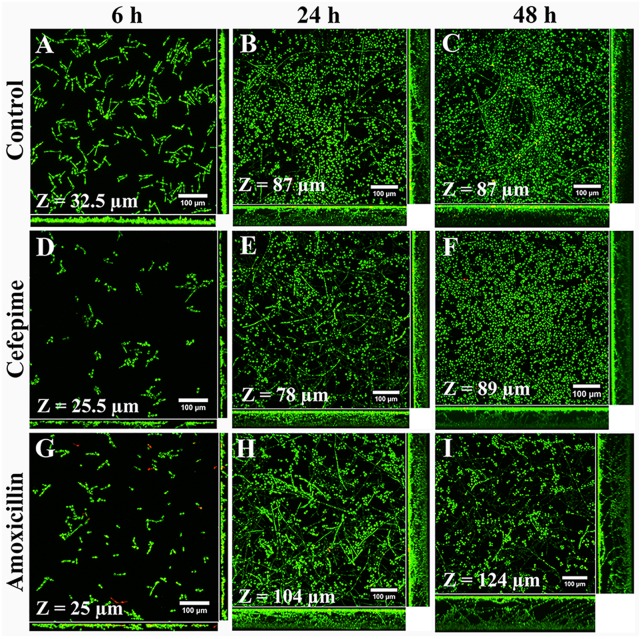
Confocal laser scanning microscopy images of *C. albicans* ATCC 10231 biofilms formed in RPMI medium without antibiotics **(A–C)** or supplemented with CEF **(D–F)** or AMOX **(G–I)**. Magnification: 400×.

**FIGURE 4:**
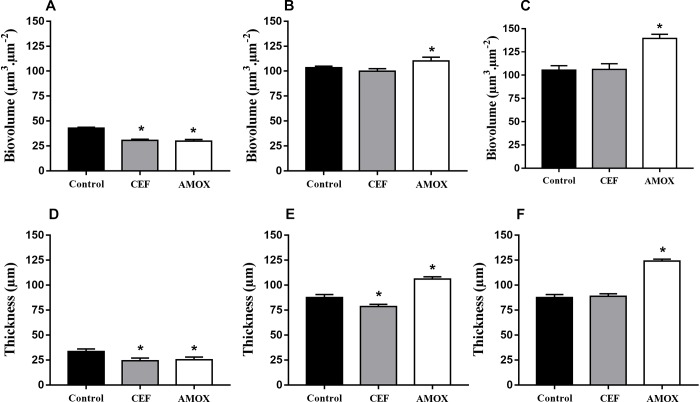
Effect of CEF and AMOX on biovolume and thickness of *C. albicans* ATCC 10231 biofilms analyzed by confocal laser scanning microscopy during adhesion at 6 h **(A,D)**, development at 24 h **(B,E)**, and maturation at 48 h **(C,F)**. Black bars: drug-free controls; gray bars: treatment with CEF; white bars: treatment with AMOX. ^∗^Statistically significant differences compared to the respective drug-free control (*P* < 0.05).

Structural differences between biofilms grown in RPMI medium with antibiotics and controls (Figure 3A–C) were better seen by confocal microscopy. CLSM showed that CEF and AMOX reduced both biovolume and thickness (Figure 3D,G and Figure 4A,D) of 6h-grown biofilms (*P* < 0.05). However, following this stage, sessile cells were stimulated by AMOX, showing increased biovolume and thickness (Figure 3H,I and Figure 4B,C,E,F) in both 24 h and 48 h-grown biofilms (*P* < 0.05). Biofilms treated with CEF showed only slight reduction in thickness (Figure 4E) at 24 h (*P* < 0.05); 24 h and 48 h-grown biofilms were similar to controls (Figure 3E,F).

**FIGURE 5:**
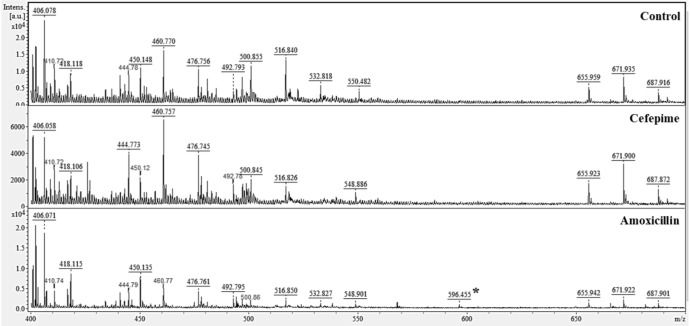
Effect of CEF and AMOX on the protein composition of the biofilm matrix of *C. albicans* ATCC 10231 analyzed by matrix-assisted laser desorption/ionization time-of-flight mass spectrometry (MALDI-TOF MS). ^∗^596.455 Da identified as Hsp70.

Matrix analysis of *C. albicans* ATCC 10231 biofilms treated with CEF or AMOX by MALDI-TOF MS (Figure 5) showed a group of peaks with *m*/*z* of 399.95–714.30 Da. Comparative analysis of the mass spectra in the mMass software showed that treatment with CEF suppressed the peak of 532.818 Da, which was seen in the matrix of untreated biofilm. However, treatment with AMOX led to the observation of a 596.455-Da peak. MS/MS analysis of this peak after fragmentation was identified by the SearchGUI software as a heat shock chaperone Hsp70 located on Gene KAR2, as indicated by *C. albicans* ATCC MYA-2876 database (protein access code in UniProtKB: A0A1D8PG96).

**FIGURE 6:**
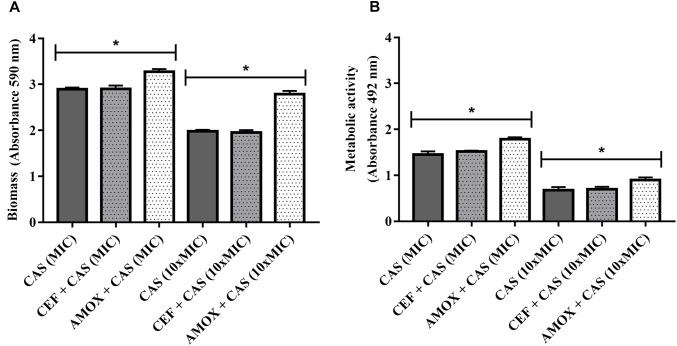
Effect of CEF and AMOX on caspofungin (CAS) susceptibility of *C. albicans* ATCC 10231 biofilms evaluated by biomass **(A)** and metabolic activity **(B)**. MIC: minimum inhibitory concentration; dark gray bars: treatment with CAS. Dotted bars: treatment with antibacterials and CAS. ^∗^Statistically significant differences between groups (*P* < 0.05).

Finally, biofilms formed in the presence of AMOX became more tolerant to caspofungin at both MIC and 10 × MIC, as shown by the increase in biomass and metabolic activity (Figure 6A,B) (*P* < 0.05). Treatment with CEF or AMOX did not modify the tolerance of *C. albicans* ATCC 10231 biofilms to amphotericin B and azoles (*P* < 0.05) (data not shown).

## Discussion

Although it is clear that antibiotics indirectly promote the overgrowth of *C. albicans* on mucosae (Samonis et al., 2006a,b, 2013), it is unknown whether these drugs can directly alter fungal metabolism (Vogel et al., 2008, 2013). In this context, even though β-lactams are one of the most widely used antibacterial drugs in medicine, there are no studies on their effects on *C. albicans* metabolism and virulence.

Cefepime (a semisynthetic, fourth-generation cephalosporin) and AMOX (a semisynthetic derivative of penicillin) are β-lactam antibiotics with antibacterial activity. Both drugs inactivate penicillin-binding-proteins – the enzymes involved in the assembly of the bacterial cell wall – and its re-organization during cell division. Although bacterial cell wall components are exclusively found in prokaryotic cells, it is now recognized that beta-lactam antibiotics interfere with eukaryotic metabolism, by inducing gene expression in neuronal cells (Rothstein et al., 2005) and modulating T cell functions after down-regulation or up-regulation of several genes (Mor and Cohen, 2013). In a previous study, Do et al. (1987) showed that high concentrations of beta-lactams inhibit the *in vitro* growth of human cells by inhibiting DNA polymerase alpha. Although we did not study the molecular targets of CEF and AMOX in *C. albicans* cells, it is reasonable to suppose that these drugs may also interact with fungal DNA polymerases, hence interfering with DNA replication. Therefore, beta-lactams may upregulate the expression of several genes, including those related to growth and virulence in *C. albicans*.

It has been previously shown that β-lactam antibiotics and vancomycin stimulate the planktonic growth and virulence of *Candida* spp. (Cordeiro et al., 2018). In the present study, we showed that β-lactams also stimulate *C. albicans* biofilms, by enhancing biomass production and metabolic activity and increasing the number of viable cells. In addition, AMOX increased biovolume and thickness of mature *C. albicans* biofilms. The thickness of the mature biofilms is the result of several factors that make this community structurally more complex. Part of this process is associated with the production of an exopolymeric matrix, where yeasts and hyphae are encased, forming a complex network with high enzymatic activity for the extracellular digestion of nutrients (Soll and Daniels, 2016; Cavalheiro and Teixeira, 2018). In the present study, antibiotic-exposed biofilms were thicker than unexposed growth control. These findings are particularly important as biofilm thickness may be related to increased antifungal tolerance (Al-Fattani and Douglas, 2006).

It was also observed that AMOX-exposed biofilms showed a substantially higher proteolytic activity than drug-free controls. It is well known that proteolytic activity is fundamental for *in vivo C. albicans* growth, as it favors adhesion, penetration into host tissues, and establishment of infection (Polke et al., 2015). The major proteolytic enzymes produced by *C. albicans* are secreted aspartic proteases, which can degrade a large number of cellular substrates, including host tissue structural proteins such as collagen, as well as immunoglobulins and complement proteins (Polke et al., 2015). These enzymes are also produced by sessile communities and have been related to biofilm adhesion, development, cell–cell communication, and matrix production (Winter et al., 2016).

Based on the increased metabolic activity and structural complexity of *C. albicans* ATCC 10231 biofilm after exposure to antibiotics, we investigated the effect of these antibacterials on the protein composition of the exopolymeric matrix. It was seen that AMOX induced the synthesis of a heat shock chaperone of 70 Da denoted as Hsp70. Heat shock proteins are highly conserved molecules in eukaryotes and prokaryotes, participating in several cellular metabolic pathways, and their main function is to help other proteins to maintain their three-dimensional conformation while remaining active (Gong et al., 2017). Six types of Hsp (Hsp104, Hsp90, Hsp70, Hsp60, Hsp21, and Hsp12) have been described in *C. albicans*, but in general, Hsp90 and Hsp70 are the most studied molecules in fungal pathogens (Gong et al., 2017). Hsp proteins potentiate a rapid evolution of antifungal resistance in planktonic cells (Cowen and Lindquist, 2005) and participate in the dispersion and antifungal resistance of biofilms (Robbins et al., 2011). In the present study, it was shown that AMOX increased the amount of chitin produced by *C. albicans* ATCC 10231 biofilm. Chitin is a minor structural carbohydrate presented in cell wall and septa and is essential for cell division, hyphal growth, and virulence (Ruiz-Herrera et al., 2006). Previous studies have shown that fungal chitin has an important anti-inflammatory function, blocking the recognition of *C. albicans* by macrophages (Mora-Montes et al., 2011) and inducing the secretion of IL-10 (Wagener et al., 2014). In addition, purified chitin reduces NO production by macrophages by increasing arginase 1 (Wagener et al., 2017). The results presented herein lead to the assumption that the AMOX-induced increase in chitin may also enhance the virulence of *C. albicans* sessile cells.

As antifungal resistance is a hallmark of fungal biofilms, we investigated if beta-lactam-exposed biofilms were more tolerant to antifungals. We found that AMOX-exposed biofilms are more tolerant to caspofungin, which may be related to the increase in chitin contents. A study conducted by Lee et al. (2012) showed that the high amount of chitin in *C. albicans* cell wall confers *in vivo* resistance to caspofungin. Therefore, our results corroborate the link between elevated chitin contents and reduced caspofungin susceptibility. We suppose that a similar phenomenon could occur *in vivo*, during therapy with AMOX.

Some limitations of our study need to be addressed. First of all, the study was conducted with only one strain, and although the experiments were conducted in replicates, intraspecific variations among strains of the same species must be considered. Differences in the response to different β-lactam drugs were seen; therefore, the effect of other antibiotics on *C. albicans* must be evaluated.

We have demonstrated the phenotypical effect of CEF and AMOX on *C. albicans* ATCC 10231 biofilm growth and caspofungin tolerance. Although both antibiotics showed stimulating effect, AMOX induced more significant changes in *C. albicans* biofilms than CEF. However, several key questions from this work remain unanswered. In this context, further studies must be performed in order to characterize the expression levels of genes related to biofilm adhesion and development, as well as virulence-related genes of *C. albicans* biofilms, after exposure to beta-lactams. The increase in chitin content in antibiotic-exposed biofilms also requires further studies with immune cells, as this polysaccharide hampers the inflammatory response. The results of the present study suggest a possible route for the complex pathogenesis of opportunistic candidemia in antibiotic-treated patients.

## Author Contributions

RC, AE, JS, DM, and MR designed the research. AE, RS, AA, PM, JO, LA, VP, and RL-N performed the experiments. RC, AE, DM, and MR analyzed the data. RC, AE, DM, and MR wrote the manuscript. RB and JS critically revised the manuscript.

## Conflict of Interest Statement

The authors declare that the research was conducted in the absence of any commercial or financial relationships that could be construed as a potential conflict of interest.

## Footnotes

1 www.mmass.org

2 http://www.uniprot.org

3 http://www.uniprot.org/batch/
